# Protocol for the *Mason: Health Starts Here* prospective cohort study of young adult college students

**DOI:** 10.1186/s12889-021-10969-5

**Published:** 2021-05-12

**Authors:** Alison E. Cuellar, Leah M. Adams, Lilian de Jonge, Virginia Espina, Laurette Espinoza, Sarah F. Fischer, Cara L. Frankenfeld, Denise A. Hines, Olga Kornienko, Heidi Y. Lawrence, Ziaul H. Rana, Niloofar Ramezani, Matthew E. Rossheim, Jerome L. Short, Eric N. Waithaka, Alyssa N. Wilson, Lawrence J. Cheskin

**Affiliations:** 1 Department of Health Administration and Policy, George Mason University, Fairfax, USA; 2 Departments of Psychology, and of Women & Gender Studies, George Mason University, Fairfax, USA; 3 Department of Nutrition and Food Studies, College of Health and Human Services, George Mason University, Peterson Hall 4113, Fairfax, USA; 4 Center for Applied Proteomics and Molecular Medicine, George Mason University, Fairfax, USA; 5 Department of Psychology, George Mason University, Fairfax, USA; 6 Department of Global and Community Health, George Mason University, Fairfax, USA; 7 Department of Social Work, George Mason University, Fairfax, USA; 8 Department of English, George Mason University, Fairfax, USA; 9 Department of Statistics, George Mason University, Fairfax, USA

## Abstract

**Background:**

Young adulthood is a period of increasing independence for the 40% of young adults enrolled in U.S. colleges. Previous research indicates differences in how students’ health behaviors develop and vary by gender, race, ethnicity, and socioeconomic status. George Mason University is a state institution that enrolls a highly diverse student population, making it an ideal setting to launch a longitudinal cohort study using multiple research methods to evaluate the effects of health behaviors on physical and psychological functioning, especially during the COVID-19 pandemic.

**Results:**

*Mason: Health Starts Here* was developed as a longitudinal cohort study of successive waves of first year students that aims to improve understanding of the natural history and determinants of young adults’ physical health, mental health, and their role in college completion. The study recruits first year students who are 18 to 24 years old and able to read and understand English. All incoming first year students are recruited through various methods to participate in a longitudinal cohort for 4 years. Data collection occurs in fall and spring semesters, with online surveys conducted in both semesters and in-person clinic visits conducted in the fall. Students receive physical examinations during clinic visits and provide biospecimens (blood and saliva).

**Conclusions:**

The study will produce new knowledge to help understand the development of health-related behaviors during young adulthood. A long-term goal of the cohort study is to support the design of effective, low-cost interventions to encourage young adults’ consistent performance of healthful behaviors, improve their mental health, and improve academic performance.

## Background

The transition from adolescence to adulthood in postindustrial societies varies considerably in duration. It may be an extended and “emerging” period characterized by exploration of identity and human capital acquisition for professional and technological careers [1], or it may be a brief and “accelerated” period in assuming adult roles (e.g., completing school, working fulltime, creating an independent household, or parenting) due to marginalized identities, such as gender, ethnic/racial minority, immigration status, or poverty status [2]. The pace and processes of young adult development and maturation, notably social-cognitive and motivational systems, lay the foundation for self-efficacy, decision-making, and behavioral self-regulation [3]. Further, the transition to attendance at a university is a critical time for establishing and reinforcing healthy or unhealthy behaviors, including those related to nutrition, exercise, sleep, substance use, and mental health [4]. Approximately 40% of young adults enroll in a college or university in the US [5], making this a social context of considerable influence during this period of development and maturation. Universities are uniquely positioned to study cohorts of young adults for the assessment and promotion of health. Such research has the potential to yield insights, and guide interventions and policies that improve students’ health and educational outcomes.

Mental and physical health issues are leading factors interfering with students’ academic performance [6, 7]. Approximately 32% of US college and university students report significant mental health symptoms, including depression (17%), generalized anxiety (7%), and suicidal ideation (6%) [8]. A recent study of 1441 US adults ages 18 to 39 found that the prevalence of depressive symptoms was more than 3-fold higher during the COVID-19 pandemic compared with before the pandemic [9]. College and university students in the US engage in heavier drinking than their non-college peers [10–12]. Approximately 30% of these students ages 18 to 22 currently binge drink, and 20% used an illicit drug within the past month [13]. Additionally, disordered eating behavior, such as binge eating, excessive exercise, and restriction is prevalent among US students [14, 15] and linked to all of the mental health conditions described above [16].

By the time they enroll in a college or university, most US students have engaged in sexual behavior at some point in their lifetime [17, 18]. Rates of sexually transmitted infections (STIs) are highest among those in the traditional undergraduate students’ age range [19]. US samples of undergraduate students highlight inconsistent condom or other protective barrier use during sex [17], along with low rates of testing for STIs [20]. US undergraduates, particularly females, report very high rates of dating and intimate partner violence [21]. Studies indicate that an average of 10% of female students in the US experience at least one sexual assault during the academic year, and rates are especially high among first year students [22, 23]. One recent study of 155,026 students across 196 US campuses found that the percent of students with lifetime mental health diagnoses had increased from 22 to 36% [24]. Of importance to academic outcomes, students with mental health conditions are at higher risk of dropping out than other students [25]. All these factors underscore the need for improved understanding of emotional and behavioral health during the undergraduate years.

Unfortunately, higher rates of college enrollment have not uniformly translated into increased rates of graduation. Many students drop out or do not complete their degrees in 4 years, which creates a large economic burden nationally [26, 27].

Several factors that promote undergraduate students’ mental health and the likelihood of completing their degrees in the US have previously been identified. For example, the presence of supportive social networks across multiple systems of relationships, including peers, friends, romantic partners and other adults can support personal and academic success [28]. Recent research has linked supportive social networks to mental health [29] as well as to educational perseverance and achievement [30]. Other psychosocial factors, such as self-perceptions (self-esteem and self-efficacy), attributions (internal control and mastery), motivation (goals and values), and self-regulation (time management and study skills) predict persistence in staying in school and achievement [31]. Further, a variety of young adults’ personality strengths, such as meaning in life, hope, grit, gratitude, curiosity, and knowledge of one’s strengths have been correlated with reduced psychological symptoms and increased life satisfaction [32].

Other health behaviors known to shift substantially in young adulthood include diet and exercise, which can have considerable long-term implications. Excessive weight gain and higher rates of obesity are among the changes experienced by young adults. Obesity prevalence among young adults in the US is 34%, compared to 20% among adolescents [33]. Obesity has been found to be a substantial risk factor for, among many other conditions, type-2 diabetes, heart disease, severe osteoarthritis, and mortality [33]. Moreover, this risk is disproportionately experienced by select racial and ethnic groups [30]. While under-researched, ethnic disparities in the prevalence of obesity and excessive weight gain may begin during young adulthood [34–36]. Particularly relevant for US college and university campuses is that obesity has been found to spread along social and family networks [37]. Fortunately, the risk of developing obesity, associated co-morbidities, and their attendant costs may be substantially reduced through even modest (5–10%) weight loss. Modest weight loss can reduce diabetes risk up to 58% in individuals with pre-diabetes---a finding consistent for at least 15 years---as well as reduce diabetes-related medical complications [38–40].

Rates of physical activity fall sharply in the US when young adults transition from high school to undergraduate years [41]. This decline is associated with a variety of factors, from psychosocial, to levels of self-efficacy, and to perceived health benefits. Despite the health benefits of physical activity, among them improved cognitive abilities and lower stress, college students face many demands on their time, including academics and work. Previous studies in the US have found that students with higher fitness levels have lower credit loads and study less than students with lower fitness levels, underscoring the trade-offs that students may face [42]. Students’ physical activity is related to better academic performance. Data from US university and college students has found that adequate physical activity, sleep, and diet were positive predictors of grade point averages [43], which opens the doors to a variety of novel studies and interventions that can target not just health but also academic and employment outcomes. Numerous questions remain among young adults, including the role of social media exposure in physical activity, differences in physical activity levels by socioeconomic status, ethnicity, culture, gender and other health factors, and whether low-cost, effective interventions can be designed to encourage participation in exercise, improved eating habits, improved academic performance and improved mental health.

Overall, previous evidence points to the importance of capturing the undergraduate experience early on, because experiences during the first year are predictive of continued enrollment in post-secondary education [44]. For example, in the US, excess stress during the first semester has been found to be a predictor of poor academic performance, and further, poor academic performance in the first year predicts attrition [45]. Further, it has been observed that many maladaptive health behavior patterns begin in the first year and remain stable throughout later undergraduate years [46, 47].

In contrast to the many other studies of young adults which, like those cited above, focus on specific aspects of students’ behaviors and health, the *Mason: Health Starts Here* was developed as a longitudinal cohort study to examine a broad range of physical health, mental health, and undergraduate degree completion determinants among successive waves of young adults who are first-time, first year university students. The overall goal is thus to improve understanding of the natural history and trajectories of young adults’ behaviors and health during undergraduate years. Of particular interest and utility would be to better characterize factors which increase the likelihood of current and future problems (‘risk factors’), or reduce those risks (‘protective factors’). It is anticipated that the study will produce new knowledge to support the design of low-cost interventions to encourage adoption of healthful behaviors, improve mental health, and improve academic performance among young adults.

## Methods

### Objectives and study design

*Mason: Health Starts Here* is a longitudinal cohort study of first-time, first year university students. Its objectives are:

- to examine health, health behavior, and mental health as predictors of college completion;
- to examine the applicability of emerging adulthood or accelerated adulthood theories to college students who differ by socioeconomic status, sexual orientation, ethnicity, culture, gender, and other demographic factors;
- to examine nutrition and physical activity levels during the college years, a time of increasing adult independence
- to examine differences in longitudinal associations among physical and psychological health factors by socioeconomic status, sexual orientation, ethnicity, culture, gender, and other demographic factors;
- to examine how social network influences and social media modulate healthful and unhealthful behaviors;
- to examine how social connectedness and trauma exposures may impact mental and physical health;
- to examine how current and past risky behaviors, sexual risk, gun ownership, and substance use modulate health, mental health, and successful college completion; and
- to identify modifiable risk and protective factors to inform new interventions to improve health and well-being.

**Fig. 1:**
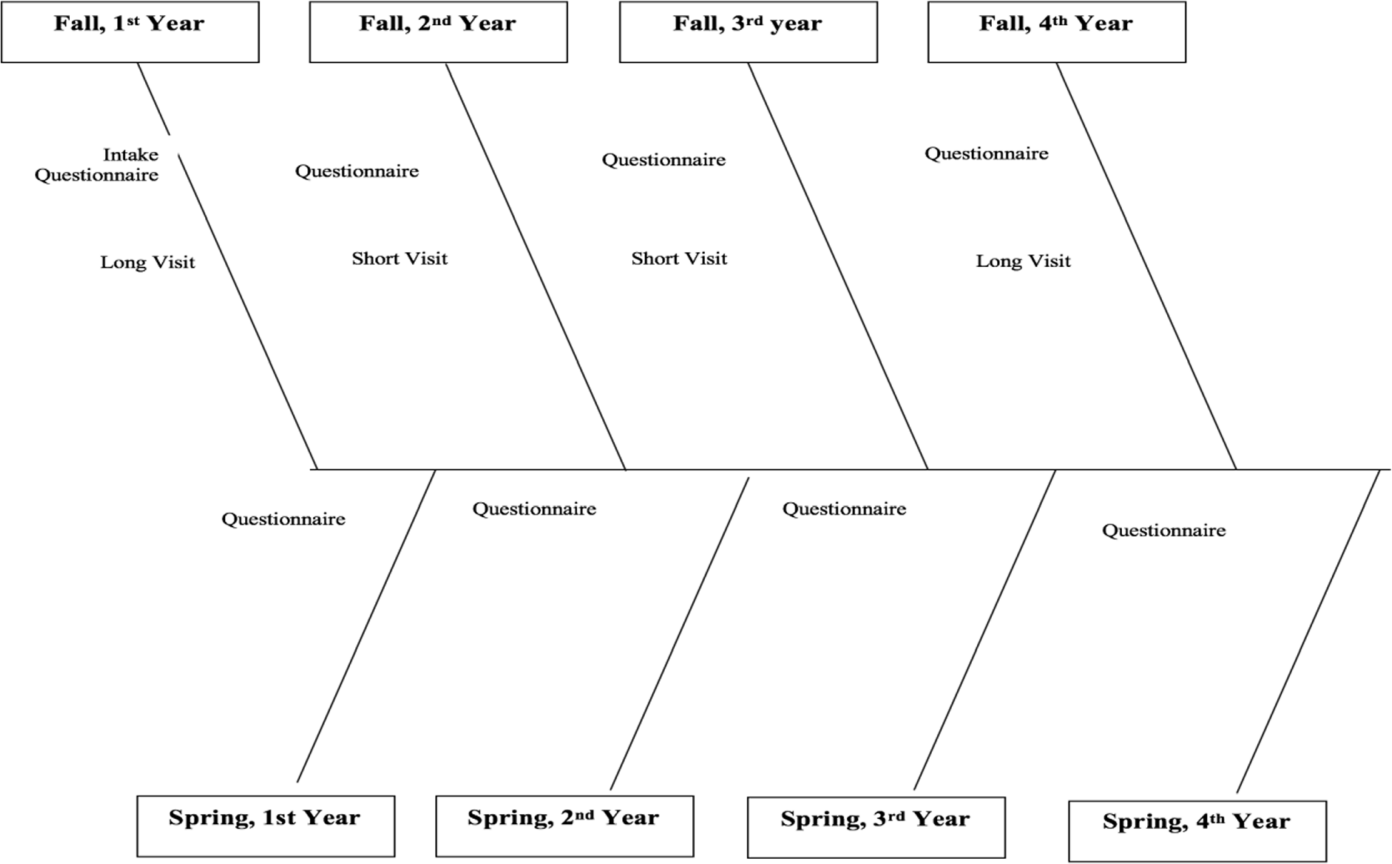
Schema of recruitment times, questionnaire administration, and study visits for the full study

Data collection occurs each fall and spring semester, and each cohort is followed for 4 years. Each fall and spring semester, participants will be contacted to participate in online questionnaires. Each fall, they will also be asked to participate in an in-person physical exam in a research center which includes biospecimen collection. Saliva will be collected in all 4 years, and blood will only be collected during years 1 and 4. The longitudinal capture of data is illustrated in Fig. 1, along with timing of incentives.

### Study population

The study includes first year students who are 18 to 24 years old who are able to read and understand English. First year students are defined as students who are newly embarking on a four-year undergraduate degree at George Mason University. The goal is to recruit 300 participants each year.

#### Study hypothesis, power calculation and data analysis

While a number of hypotheses will be tested, the power calculation was based on the hypothesis that alcohol consumption predicts student drop-out. For the optimal sample size procedure, predicting students’ drop-out based on their alcohol consumption, and other factors including demographics, and using measures from previous studies [48], we will have a power of 0.8, type I error rate of 0.05, a confidence level of 95%, and a fairly small odds ratio of 1.7 to detect an approximate effect size of 0.3. The estimated sample size for examining if students are more likely to drop out of college during their first year when they have higher alcohol consumption is about 465 students, a sample size which is planned to be achieved by the end of the second year of the study. Power analysis was performed using SAS 9.4 [49].

Upon completion of the longitudinal data collection and data exploration procedures, young adults’ indicators of physical and mental health, demographic variables, and other factors such as firearm access, substance use, technology and social media use, and measures of nutrition, physical activity and sleep will be used in longitudinal models to evaluate their role in their college completion and other outcomes of interest. Depending on the research question, the following longitudinal approaches will be used to identify the factors that influence college completion and other response variables of interest over time. General linear models and generalized linear models will be fitted when the dependent variables are continuous and non-continuous, respectively. Generalized estimating equations will be used instead when distributional assumptions are not met [50]. For assessing individual changes over time, while accounting for the clustering of observations within different cohorts, colleges, or any other cluster which results in similar behavior (i.e. hierarchical data), a random intercept growth hierarchical linear model will be fitted [51].

Performing such models and analyzing the trend of events over time allows us to gain an insight into students’ physical and mental well-being while they complete their college education [52]. Additionally, these modeling approaches will result in robust model selections and inferences [53]. Within these models, we will correct for the “cohort effect”—that is allowing for analysis of the individual time components of cohort (range of birth dates or when they started college), period (current time), and age (at point of measurement)—and will account for the impact of each individually.

### Recruitment and retention

We are using a purposive, convenience sample that is being recruited to have similar demographic characteristics to the university’s diverse student population. In the pilot year (academic year 2019–2020), the study was advertised to students in selected courses with large numbers of first year enrollees through flyers outside of classrooms, brief in-class presentations and to select student organization, online video, postcards distributed in class, and email. Beginning in 2020, contact information of incoming first year students was obtained from University Life and recruitment was expanded to all incoming first year students reached through invitation letters and emails, along with video and information during first year orientation sessions, and messages to followers on University social media. Additional recruitment occurs by asking faculty teaching courses with high proportions of first year students to share the recruitment video with their classes. Because of COVID-19, in 2020–2021 all recruitment is virtual, but once a higher proportion of in-person classes and student organizations resume in future years, in-person presentations about the study will resume.

Once participants complete an online survey, they are invited to make an appointment for an in-person physical exam and biospecimen collection. Participants are offered $10 for completing each online survey and an additional $25 for completing each in-person visit. Participants receive personalized health feedback based on their survey responses and physical exam. To encourage study retention, participants receive survey reminder emails and birthday emails. Participants are asked to provide contact information of friends or family to facilitate follow-up. The study team also delivers health-related messages to the participant community, such as tips for stress management, and will share summary study findings when available.

#### Data collection

The study includes online and in-person data collection. Table 1 lists all study measures and the timing of data collection.

**Table 1:** Descriptions, points of data collection, and references of the measures included in the study

	Baseline Only	Fall	Spring	Citation	# of items
**Online Survey**					
Demographics		X	X		
Financial Support		X		[54]	1
Disability/ability Status		X			1
Anger, Depression, Anxiety		X	X	[55]	21
Loneliness		X	X	[56]	4
PTSD		X	X	[57]	20
Alcohol Use Disorders		X	X	[58]	10
30 Day Alcohol Use NSDUH		X	X	[59]	1
30 Day Binge Drinking NSDUH/BRFSS		X	X	[59, 60]	1
30 Day Drinking Max BRFSS		X	X	[60]	1
30 Day Drinking and Driving YRBS/NSDUH		X	X	[59, 61]	1
30 Day Driving Under Influence YRBS/NSDUH		X	X	[59, 61]	1
Riding with Drinking Driver YRBS		X	X	[61]	1
Drug Use		X	X	[62]	10
Vaping E-cigarette		X	X	[60]	2
Cigarette Smoking		X	X	[11]	1
Cigarette Smoking, 30 Days		X	X	[59]	1
Cigarette Menthol, 30 Days		X	X	[59]	1
Marijuana Use		X	X	[60]	3
Eating Disorders		X	X	[63]	31
Rumination		X		[64]	4
Perceived Stress		X	X	[65]	4
Migraine Severity	X			[66, 67]	14
Life Satisfaction		X	X	[68]	5
Happiness		X	X	[69]	4
Self-Rated Health		X	X	[70]	1
Life Events Checklist, Baseline and Follow-up	X		X	[71]	17, 9
Sexual History, Contraceptive Use, Perceived Risk, and PrEP Knowledge	X		X	[72–76]	42, 2, 3, 4
Sexual Experiences, Baseline and Follow-up	X		X	[77]	10
Adverse Childhood Experiences	X			[78]	10
Dating Violence		X		[61]	9
Online Dating Violence		X		[79]	2
Everyday Inequity		X	X	[80]	6
Ethnic Identity Scale, Baseline	X		X	[81]	9
Language Brokering Scale	X			[82]	1
Social Connectedness		X		[83]	10
Impulsivity/Impulse Control		X		[84]	21
Physical Activity		X	X	[85]	7
Texting/e-mailing While Driving		X		[61]	1
Sleep Quality		X	X	[86]	24
Gratitude		X	X	[87]	6
Hope		X	X	[88]	8
Meaning in Life		X	X	[89]	5
Perceived Social Support		X	X	[90]	12
Mini Personality Scale		X		[91]	20
Personal Network/Social Structure		X	X	[92, 93]	23
USDA + Personalized Feedback		X		[94]	4
Food/Personal Care Products		X		[95]	44
Sustainable Consumption Behavior Nutrition		X		[96]	18
New Environmental Paradigm		X		[97]	15
Food Insecurity		X		[98]	2
Housing Security		X		[99]	2
Past and Anticipated Voting		X			7
Technology Use		X	X	[100]	11
Online Status Indicators		X		[101]	4
Social Media Integration Scale		X		[102]	6
Night-Time Social Media Use		X	X	[103]	7
DHQ-III Food Frequency		X		[104]	135
Self-Compassion			X	[105]	26
Compassion			X	[106]	16
**In-Person Clinic Visit**					
Health History Form	X				
Physical Exam (body weight, height, waist circumference, blood pressure, pulse, respiratory rate, current oral temperature, heart and lung exam, abdominal exam, neck exam, and general physical appearance)	X				
Self-Injurious Thoughts and Behaviors (SITBI)	X			[107]	25
Saliva Collection		X			
Blood Collection	X				
Urine Collection	X				
Body Composition via DXA Scan	X				
Resting Metabolic Rate via Fitmate	X				
Stool Kit Collection (Optional)	X				

### Online questionnaire

The online data collection focuses on physical and emotional health, nutrition, sleep, civic engagement, and academic success. Note that some instruments/measures have not been evaluated specifically in 18–24 year olds, which the current study will help researchers to assess.

#### Demographics and social determinants of health

These items include age, race, ethnicity, mother’s and father’s education, current employment status and hours worked, whether the participant lives on campus, romantic relationship status, religious preference, country of birth for participant and parents, sexual identity, current gender, and gender at birth. *Adult food security* was assessed using two items adapted from the Hunger Vital Sign screener [98]: “1) I worried about not having enough to eat and 2) I tried not to eat a lot so that the food would last.” The response options are: often true, sometimes true, never true. *Housing insecurity* was assessed using two items adapted from the Veterans Screener [99]: “In the past 2 months, have you been living in stable housing that you own, rent, or stay in as part of a household?” and “Are you worried or concerned that in the next 2 months you may NOT have stable housing that you own, rent, or stay in as part of a household?” Both questions are answered with a yes or no.

#### Health

*Self-rated health* (SRH) is measured with one item that asks, “Would you say your health is excellent, very good, good, fair, or poor?” Responses range from 1 (poor) to 5 (excellent). SRH is strongly correlated with objective health status [70].

#### Migraine

The presence of migraine and migraine severity is assessed using the American Migraine Prevalence and Prevention (AMPP) diagnostic module (7 items) [66] and the Migraine Disability Assessment (MIDAS) questionnaire (7 items) [67], respectively.

#### Nutrition, physical activity, and sleep

Respondents are asked to self-report height and weight. Habitual dietary and supplement intake is assessed using the Diet History Questionnaire (DHQ)-III food frequency questionnaire, a 161-item tool which asks about food intake over the past month [108]. *Physical activity* is measured with the International Physical Activity Questionnaire (IPAQ) - short version, a 7-item questionnaire [109]. *Sleep quality* is measured with 24 items from the Pittsburgh Sleep Quality Index or PSQI [86]. The PSQI items form seven component scores that range from 0 to 3 points. Some items are fill-in-the blank and others have four choices to measure sleep quality, latency, duration, efficiency, disturbances, medication, and dysfunction during the past month. The scale has a test-retest reliability of 0.87 overall, and 0.53 to 0.81 for the different components [110].

#### Technology use and social media

Technology Use is measured with questions about 10 types of social media and amount of use with a 10-point response scale [100]. Online Status Indicators [101] are measured with 4 questions with a 5-point response scale of how important number of followers and likes are on social media to them [101]. The Social Media Integration Subscale [102] consists of 6 questions with a 5-point response scale. To assess emotional investment in social media, a modification of the Social Integration and Emotional Connection subscale of the Social Media Use Integration Scale [102] will be used. Nighttime-specific Social Media is measured with seven items rated from Never [1] to Daily [7].

#### Perceived risk of sexually transmitted infection, contraceptive use and sexual history

Sexual behavior and attitudes are measured with a total of 51 modified items about sexual behaviors (42 items) [72], types of contraceptives (2 items) [73], perceived risk and susceptibility to sexually transmitted infections (3 items) [74], and PrEP knowledge (4 items) [75, 76].

The Sexual Experiences Survey (SES) [77] asks participants whether or not they have experienced unwanted sexual contact via a number of different means. The questionnaire has been modified to ask participants if they have experienced each type of unwanted sexual assault since the age of 14 and in the past 3 months. Participants respond ‘yes’ or ‘no’ to a series of 10 questions describing sexual assault experiences [77].

#### Firearms

There are 30 questions assessing a variety of firearm related topics including: acquisition, ownership, access, proximity, safe storage, training, carrying behavior, reasons for carrying, and firearm type, as well as whether they knew someone who was unintentional or intentional shot. These items were adapted from several national surveys including the National Firearm Survey, Pew Research Center, National Comorbidity Survey - Adolescent Supplement (NCS-AS), as well as from previously conducted research [111, 112].

#### Substance use

Alcohol use is measured with the Alcohol Use Disorders Identification Test (AUDIT), a 10-item scale assessing alcohol consumption, drinking behaviors, and alcohol-related problems, which is used to identify individuals at risk of an alcohol use disorder [58]. The psychometric properties of the AUDIT have been extensively examined in multiple age groups, including undergraduate drinkers [113]. Internal consistency is above .70 in these samples. Degree of Problems Related to Drug Abuse is measured using the 10-item Drug Abuse Screening Test (DAST) [62]. Similarly, the psychometric properties of the DAST are well established in multiple samples, including young adults, and internal consistency and test re-test reliabilities range from .70s to .90s [114]. Ever smoking cigarettes, past 30-day cigarette smoking, and 30-day menthol cigarette smoking items were used/adapted from the Monitoring the Future (MTF) study and National Survey on Drug Use and Health (NSDUH). Vaping/electronic cigarette (e-cigarette) use was measured using items from the MTF study, modified with nicotine clarification as part of the instructions per the Behavioral Risk Factor Surveillance System (BRFSS).

Past 30-day alcohol consumption items were measured using or adapting items from the NSDUH and BRFSS. Marijuana use was measured using three items from the BRFSS: ever use (yes/no), past 30-day use frequency, and manner of use (i.e., smoke, eat, drink, vaporize, dab, don’t know, unsure). Risky driving-related behaviors (i.e., driving under the influence of alcohol, riding in a vehicle driven by someone who had been drinking, driving under the influence of marijuana, and texting or e-mailing while driving) were assessed using items from the NSDUH and YRBSS or adapting them (i.e., past 30-day rather than 12 month).

#### Access to health care

Health psychiatric medication, history of mental health treatment, health insurance, regular source of care and barriers to access. These are measured by “If you have ever been prescribed or currently prescribed psychiatric medicine” with a “Yes” or “No.”

#### Psychological symptoms

Eating Disorder risk and symptoms are measured with the Eating Pathology Symptoms Inventory (EPSI) [63]. The EPSI is a 45-item multidimensional measure of eating pathology and includes items related to body dissatisfaction as well as restricted eating and binge eating. Internal consistency for the subscales ranges from .84–.89; the measure has been validated in large samples of undergraduate men and women [115].

Anger, Depression, and Anxiety symptoms are measured with the Patient Reported Outcomes Measurement Information System (PROMIS) [116]. The PROMIS assesses individual functioning across various domains of well-being and has been extensively validated in multiple age groups (including young adults) as part of a large multi-year initiative by the National Institute of Health to develop a patient reported outcome measurement system and assessment center. Respondents report their feelings of these emotions over the past 7 days using a 5-point Likert scale (‘Never’ to ‘Always’). Internal consistency in a large undergraduate sample is high (depression = .94, anxiety = .92, and anger = 89).

Loneliness is measured with 4 items from the Short Scale for Measuring Loneliness measure [56]. Items are scored on a 3-point scale from “hardly ever” to “often,” with an internal consistency of .72. The short form is highly correlated with the long form of this measure [56], and has demonstrated predictive and concurrent validity in young adults and undergraduates [117].

Post-Traumatic Stress Disorder symptoms are measured with 20 items rated from 0 (not at all) to 4 (extremely) using the Posttraumatic Stress Disorder Checklist for DSM-5 (PCL-5) [57]. The PCL-5 has been validated in undergraduate and military samples [57, 118] with internal consistency reported as high as .95 in undergraduates.

Impulsivity/Impulse control is measured with the 56 item UPPS-P [84]. The UPPS-P consists of five scales assessing conscientiousness, lack of planning, sensation seeking, and acting impulsivity during periods of positive and negative mood. Participants are asked to rate their typical behavior on a scale from 1 to 5. The UPPS-P was initially developed and validated with young adult and undergraduate samples and its’ psychometric properties have been extensively tested in young adults and clinical populations. Internal consistency of the subscales typically ranges from the .80s to the .90s [84, 119].

#### Psychological well-being

Life Satisfaction is measured with 5 items from the Satisfaction with Life Scale (SWLS) [68]. Items are rated on 7-point scale from ‘strongly disagree’ to ‘strongly agree.’ The SWLS is a single factor with an alpha coefficient of .87 and test-retest reliability of .82 [68].

- Happiness is measured with 4 items from the widely used Subjective Happiness Scale (SHS) [69]. Internal consistency typically ranges from .74 to .94. The items are rated on 7-point scales with various response choices.
- Gratitude is measured with 6 items from the Gratitude Questionnaire [87]. The items are rated on 7-point scale from ‘strongly disagree’ to ‘strongly agree.’ McCullough et al. [87] reported an alpha coefficient of .82 for this measure.
- Hope is measured with the 8-item Adult Hope Scale [88]. The AHS measures two facets of hope: Agency, or goal-directed energy, and Pathways, or planning to meet goals. Items are rated on an 8-point scale from ‘definitely false’ to ‘definitely true.’ Goodman et al. [32] reported an alpha coefficient of .87 for this measure.
- Meaning in Life is measured with 5 items from the presence subscale of the Meaning in Life Questionnaire [89]. Items are rated on a 7-point scale from ‘absolutely untrue’ to ‘absolutely true.’ Goodman et al. [32] reported an alpha coefficient of .90 for this measure.
- The Ethnic Identity Scale – Brief (EIS-B) [81] consists of 9 items that examine three different domains of ethnic identity formation: exploration (3 items), resolution (3 items), and affirmation (3 items). Responses are coded so that higher scores on each subscale indicate greater exploration, resolution, and affirmation. Internal consistency has been shown for subscales, and ranged from .70 to .89 [81]. The three-factor structure has been found with Latino adolescents, and an ethnically diverse (majority and minority) sample of college students [83, 120].
- The Compassion Scale [106] and the short form of the Self-Compassion Scale [121] are measured with 16 items, and 12 items, respectively, of how you feel and behave (on a scale from 1 ‘Almost Never’ to 5 ‘Almost Always.’)

#### Psychological risk variables

The Life Events Checklist [71] is a 17-item measure asking participants to check experiences related to any of 17 potentially-traumatic events (e.g. natural disaster, accident, combat exposure) listed. Participants are asked to check one or more of the following statements: ‘Happened to me’; ‘Witnessed it’; ‘Learned about it’; ‘Not sure’; and ‘Doesn’t apply’.

Adverse Childhood Experiences assesses all types of abuse, neglect, and other potentially traumatic experiences that may be experienced by people under the age of 18 by asking participants to respond “yes” or “no” to the occurrence of 10 events in two categories: abuse (psychological, physical, and sexual) and household dysfunction (substance use, mental illness, physical violence, and criminal behavior). The prevalence of exposure is computed by summing across the abuse and household dysfunction categories [78].

Everyday Inequity Scale is a 5-item scale asking participants to report frequency, from ‘never’ to ‘at least once a week’, and reasons for experiencing racial and nonracial discrimination in their day-to-day life [80, 122]. Internal consistency for this scale is .77.

#### Peers and social support

Perceived Social Support is measured with 12 items from the Multidimensional Scale of Perceived Social Support [90]. The items are rated on a 5-point scale from 1 (strongly disagree) to 5 (strongly agree) for relationships with family, friends, and a significant other. The overall alpha coefficient is .85 and ranges from .72 to .85 for the three types of relationships [90].

##### Personal network composition and structure

Individuals report on up to 5 members of their personal networks with whom they discuss important matters. For each of these members they provide information on individual characteristics (e.g., gender, race/ethnicity, education, nature of relationship, frequency and mode of communication), rated positive and negative quality of their relationship with each network member, and estimated likelihood that network members know one another. Similar assessments have been used in large-scale aging surveys, such as the National Social Life, Health, and Aging Project (NSHAP) [92] and Chicago Health, Aging, and Social Relations Study (CHASRS) [93].

##### Personality traits

The Mini-IPIP [91] is a 20-item short form of the 50-item International Personality Item Pool-Five-Factor Model Measure [123]. The scale measures extraversion, agreeableness, conscientiousness, neuroticism, and intellect/imagination, with 4 items for each trait. The items are rated on a 5-point scale from 1 (very inaccurate) to 5 (very accurate).

#### Environment and sustainability

Environmental orientation is assessed with the 4-item revised New Environmental Paradigm Scale (NAPS) [97]. Sustainable consumption is measured with the 18-item Sustainable Consumption Behavior Scale (SCBS) [124]. The NAPS is used to uncover attitudes and beliefs in relation to the environment, while the SCBS measures what, if any, sustainable practices individuals are currently utilizing.

#### Environmental exposures

Exposures related to food, employment and personal care products [124] includes 12 items related to purchases of food and personal care items and their packaging, 2 items related to second-hand smoke, 3 questions related to type of work, and 3 items related to products used in the home.

### Assessment in the research center (in-person visit)

During the in-person visit to the population health clinic, study participants are asked additional questions about their medical history, given a history and physical exam by a clinician, and asked to donate a venous blood sample and a saliva sample. Participants are requested to fast prior to their appointment and to avoid taking medications and engaging in rigorous physical activity within an hour of their appointment.

At intake, participants are asked for medical history and family history using a checklist of conditions, including indictors of cardiovascular-related health issues, such as high blood pressure, heart disease, stroke, high cholesterol, chest pain, and irregular heartbeat. Participants also complete a past hospitalizations list, a weight history by age, a current medications list, two items on perceived problematic gambling and shopping; six questions on meal timing and meal frequency; and a source and frequency of purchased meals checklist; and two questions on wake-up time that day and participants’ usual wake-up time. Women are asked to report on a 15-item menstrual history. Participants are also asked to complete an interviewer-guided Self-Injurious Thoughts and Behaviors Interview (SITBI). This is a 25-item instrument that queries lifetime and past week self-injurious thoughts and behaviors [107]. The physical exam includes body weight, height, waist circumference, blood pressure, pulse, respiratory rate, current oral temperature, heart and lung exam, abdominal exam, neck exam, and general physical appearance. Body composition is measured using a DXA scanner (DXA, Hologic Horizon densitometer; Hologic, Boston MA). Resting metabolic rate is measured using indirect calorimeter (FitMatePro, Cosmed, Rome, Italy).

#### Biospecimens

Participants are asked to fill two cryovials with approximately 1 mL of saliva using passive drool procedures. Blood samples include a venous blood draw of 36 mL. Samples are tested for HbA1c, lipid panels, and blood chemistries. Abnormal results are communicated to participants by clinical staff. The remaining sample is processed, aliquoted, and frozen at − 70 degrees C.

## Discussion

The purpose of the Mason Cohort study is to launch a longitudinal study using multiple research methods in a racially and ethnically diverse population of young adults to evaluate the effects of health behaviors on physical and psychological functioning, especially during the COVID-19 pandemic. The study will produce new knowledge to help understand the development of health-related behaviors during young adulthood. A long-term goal is to support the design of effective, low-cost interventions to encourage young adults’ consistent performance of healthful behaviors, improve their mental health, and improve academic performance and completion of undergraduate degrees.

The Mason Cohort was thus envisioned as also being a tool to potentially benefit participants directly, in addition to benefitting our understanding as researchers in health fields. Thus, it is a hybrid design, part traditional cohort study and part intervention trial.

This has obvious benefits to participants, as information and advice concerning their own health and risk factors are returned to them, and interest in improving those risks is potentially fostered. The study design also encourages researchers, stimulated by data gathered and associations discerned to propose sub-studies, which, upon review and approval by the study steering committee are submitted for IRB review and offered to all or subsets of cohort participants based on the specific questions being asked in the sub-study. Preference will be given to proposed sub-studies which offer a direct potential benefit to study participants.

While most longitudinal cohort studies are by design, purely observational, and thus, at best of indirect benefit to participants, even these may show expectancy effects. For example, participants may be triggered to answer survey questions “appropriately” (so-called “reactivity” effects), or to address unfavorable health-related behaviors through merely being asked about them [125].

From an evaluation of theories on college student retention and success, interactionalist theory of social and academic integration suggests that college completion relies upon the extent to which students are committed to their institution, and this commitment level depends on the level of social and academic integration, determined by the quality of interactions [126]. The student attrition model, focused more on cognitive and behavioral aspects of college completion, posits a significant role for perceptions of satisfaction and attitude impacting behavioral intentions to stay or leave [127]. Besides, results from different interventions on college students suggest that low cost and brief interventions can have a meaningful impact on long-term student outcomes [128]. Therefore, this study will follow a multi-stage developmental process for preventive interventions (following Institute of Medicine recommendations); the identification of a target problem, then the review and investigation of research that can identify protective factors and inform etiologic models, and finally, preventive interventions targeting these factors using experimental designs and intervention analyses [129].

As noted in *Methods*, we are using a purposive, convenience sample that we will recruit to have similar demographic characteristics to the university’s diverse student population. If some demographic groups are underrepresented in our initial recruitment, such as non-binary gender, ethnic/racial minority, and diverse sexual identities, we will contact various campus organizations and support groups where they are members to encourage participation in our study.

The self-selected population is a popular sampling technique in many health areas that require human subjects and is an effective sampling strategy, especially in experimental research settings [130]. Since the potential research subjects contact study staff directly, this can reduce the amount of time necessary to search for appropriate participants. In addition, the potential participants are likely to be committed to taking part in the study, which can help improve attendance and a greater willingness to provide more insight into the phenomena being studied. However, with self-selection potential research participants may exhibit an inherent bias in their characteristics and approach to the study [131].

To complete any study of living beings, it is critical to retain study participants. Participants who complete their scheduled follow-up visits within predefined visit windows will be considered as retained. Although every effort is being taken to facilitate completing each participant’s entry, mid, and exit visits, attrition can and does happen; and the retention rate can fluctuate over time and across visits [132]. To ensure continued participation and minimize attrition, this study follows the key precepts of retention and practice: facilitating participation, communicating study progress, expressing appreciation, and informing participants of study results [133].

We see the future of the study as promising- its breadth of coverage of health predictors and precursors, its diverse sample of young college students, and its flexible implementation strategies enhance its capacity to inform emerging health problems and risk factors through examination of the wealth of data and specimens being gathered.

Also of note is the benefit of having baseline specimens and surveys in a diverse sample of young adults when new health problems like COVID-19 emerge.

More work needs to be done, though, including securing sources of ongoing funding, and developing collaborations with other studies of young adults/college students. Future enhancements being considered include following participants for a time frame beyond the undergraduate years to track behavioral and health trajectories across the lifespan, and gathering more family data, including adding parents and future offspring of current participants.

## Data Availability

The datasets generated during the current study are not publicly available to ensure privacy of individual-level data, but upon reasonable request. Deidentified data are available through the corresponding author after review and approval by the Study Steering Committee.
